# Effects of SWEEPS Laser-Activated Irrigation and Diode Laser Irradiation on Void Formation and Bond Strength of a New Premixed Calcium Silicate Sealer, BioRoot Flow—An In Vitro Study

**DOI:** 10.3390/bioengineering13060675

**Published:** 2026-06-10

**Authors:** Gabrijela Kapetanović Petričević, Maša Milanović-Litre, Ivana Milanović, Marko Katić, Ivica Anić, Ivona Bago

**Affiliations:** 1 Department of Endodontics and Restorative Dental Medicine, School of Dental Medicine, University of Zagreb, 10000 Zagreb, Croatiabago@sfzg.hr (I.B.); 2 Department of Materials, Faculty of Mechanical Engineering and Naval Architecture, University of Zagreb, 10000 Zagreb, Croatia; 3 Clinic of Restorative Odontology and Endodontics, School of Dental Medicine, 11000 Belgrade, Serbia

**Keywords:** bioceramic sealer, bond strength, micro-CT, SEM, push-out test, laser-activated irrigation, SWEEPS, diode laser

## Abstract

The aim of this study was to evaluate the effect of two laser-assisted disinfection techniques on the porosity and bond strength (BS) of a new premixed calcium silicate sealer. Forty extracted human single-rooted premolars with one root canal were prepared up to 50/05. Samples were randomly assigned to the groups (*n* = 10 each): 1. shock wave-enhanced emission of photoacoustic streaming (SWEEPS) (20 mJ, 15 Hz, 0.60 W, pulse duration 25 µs), 2. diode laser (975 nm, 1.5 W), 3. conventional needle and syringe irrigation (CI), and 4. control (C), with no final irrigation protocol. Root canals were filled with a premixed calcium silicate sealer using the single-cone obturation technique. Micro-CT scans were performed after two weeks to determine the presence of voids in the filling. Dentinal discs from the middle third were prepared for push-out testing. Kruskal–Wallis and post hoc Dunn tests were used, with significance set at 5%. Micro-CT analysis detected porosity in all samples, with no significant differences among the groups (*p* > 0.05). SWEEPS showed the highest BS values (median 3.233 MPa) and outperformed CI and C (median 1.923 and 1.989 MPa) (*p* < 0.05) overall. SWEEPS enhanced the BS compared with CI. Voids were present in all experimental groups.

## 1. Introduction

Three-dimensional hermetic obturation is one of the main factors of the long-term success of endodontic treatment [1], while adequate root canal filling is proportionally correlated with the long-term dimensional stability of the root canal sealer. Nowadays, calcium silicate-based sealers (CSBSs) meet those needs and are experiencing an increase in clinical use, especially with new premixed materials, due to their simple handling. Other than their biocompatibility [2], the dimensional stability and bioactivity of CSBSs after setting is the main reason why they could potentially outperform standard epoxy-based sealers. Furthermore, the literature shows a low percentage of porosity [3] and a setting reaction achieved in the presence of moisture, finally forming hydroxyapatite at the interface and creating a chemical bond to dentin [4,5].

However, studies up to now have not shown homogeneous results when evaluating the bond strength (BS) of CSBSs and epoxy resin-based sealer. Although certain studies claim higher BS values of CSBSs [6,7], a number of studies in the last five years claim that epoxy resin-based sealers outperform CSBSs in terms of BS [8,9,10].

Since the chemical–physical properties and bioactivity of CSBS are clinically relevant, it is of interest to enhance the BS to establish the full potential of the material. According to the scientific information to date, laser-assisted protocols are emerging as a potential solution, since findings show their efficacy in irrigation fluid activation, biofilm removal, debris and vital tissue removal, and root canal filling remnant removal [11,12,13,14]. Laser-assisted protocols have also been proven to change the intracanal dentinal surface, which could potentially affect the bonding of materials, as shown in studies claiming composite bonding enhancement [15,16].

Erbium:yttrium–aluminum–garnet (Er:YAG) lasers are used in endodontic treatment with the novel mode shock wave-enhanced emission of photoacoustic streaming (SWEEPS). Due to the fact that its wavelength matches the absorption peak of water, it can be absorbed by irrigation fluids and hydroxyapatite [17]. SWEEPS was created in order to increase the efficiency of the existing PIPS mode. The main mechanism involves delivering pairs of ultra-short pulses which create bubbles. An additional second bubble accelerates the collapse of the first one, forming a violent collapse and emitting a shock wave [18]. Studies showed that SWEEPS can cause canal wall erosion [19], while a group of authors reported that hydroxyapatite crystals in mineralized dentin tissue remain intact with laser irradiation of no higher than 19.11 J/cm^2^ [20]. Furthermore, laser irradiation was noticed to improve the crystallinity of lamella hydroxyapatite and rearrange its growth orientations [20]. On the other hand, diode lasers’ antibacterial efficacy has been proven [21], but other than antimicrobial efficacy, diode laser protocols have also shown melting of intracanal dentin [22,23]. Still, the use of diode lasers has been recommended due to their low costs compared to other lasers [21].

Studies have already revealed that BS is influenced by the type of irrigation [24,25], and certain studies have shown that laser-assisted protocols [26] positively affect the adhesion and BS of CSBS to the root canal dentin walls. Nevertheless, there are still few studies examining laser-assisted protocols and, according to our knowledge, none using SWEEPS technology to enhance the BS of bioceramic sealer.

Addressing this gap, the aim of this study was to compare the BS and porosity of a premixed CSBS (BioRoot Flow, Septodont, Saint Maur Des Fosses, France) used with a single-cone obturation technique (SC) after SWEEPS-mode Er:YAG laser-activated irrigation, diode laser root canal irradiation, and conventional irrigation (CI). The null hypothesis of the study was that there would be no difference in the BS and porosity of the root canal filling with the CSBS after the different final root canal disinfection protocols.

## 2. Materials and Methods

### 2.1. Sample Selection

The protocol of this ex vivo study was approved by the local Ethics Committee (number of approval 003-01/26-05-04).

Power analysis using the chi-squared test (a = 0.05 and b = 0.95) was performed, and a minimum of eight canals per test group was determined.

Single-rooted, single-canal extracted human premolars from patients aged between 25 and 35 years old (collected at the Department of Oral Surgery School of Dental Medicine) were collected and stored in 0.1% water solution of chloramine-T trihydrate and in distilled water two hours before procedures. The inclusion criteria for the samples were as follows: one root and one round root canal, with an initial patency file corresponding to ISO size 15 or 20 (confirmed with K-file #15 or #20). The exclusion criteria were as follows: oval canals, previous endodontic treatment, calcified root canals, class II carious lesions, external resorption, and internal resorption. All collected teeth were subjected to CBCT scanning, and, out of a pool of 72 teeth, 40 samples were confirmed to have one round canal (Cranex 3DX; Soredex, Tuusula, Finland, field of view 50 × 50 mm; ENDO, 85 µm; 6.3 mA; 90 kV; 8.7 s; 450.3 mGycm2). Samples were selected and standardized to a working length of 20 mm without decoronation, but the crowns were slightly polished while preserving the proximal, buccal, and oral walls.

### 2.2. Sample Preparation

The same operator (GKP) performed root canal preparation, experimental protocols and obturation.

Traditional access cavities were prepared using a water-cooled diamond fissure No. 016 (Komet, Rock Hill, SC, USA). Canal patency was confirmed according to the inclusion criteria. The working length (WL) was determined when the K-file was visible at the apical foramen under 6.5× magnification (Orascoptic eye zoom max Dragonfly pro, Orascoptic, Madison, WI, USA), and 0.5 mm was subtracted from the measured length. The apical foramina of all selected roots were sealed with hot glue and embedded in polyvinylsiloxane (Exaflex putty, GC, Lucerne, Switzerland) to create a closed system [27].

Root canals were instrumented using a reciprocating engine-driven system up to ISO size 50/0.05 (Reciproc Blue RB50, VDW Dental, München, Germany) using a motor set at reciprocation motion (Reciproc Gold, VDW Dental, München, Germany). During the instrumentation, 5 mL of 3.5% sodium hypochlorite (NaOCL) was used per canal, using a 31-gauge needle (SteriTips, DiaDent, Burnaby, BC, Canada) and 2 mL syringe.

After the instrumentation, the root canals were dried using corresponding paper points, and the samples were randomly assigned to one of three test groups or one control group using a random number table as follows:

Group 1. SWEEPS (*n* = 10)

The samples were treated using an erbium-doped yttrium–aluminum–garnet (Er:YAG) laser (LightWalker AT, Fotona, Ljubljana, Slovenia) with a radial laser tip (600 µm, 9 mm, Fotona, Ljubljana, Slovenia). During activation, the irrigant was constantly delivered in the access cavity using a 31-gauge needle (DiaDent), and the radial laser tip was inserted into the access cavity and maintained in a fixed position. Auto SWEEPS mode parameters were used: pulse energy (20 mJ), pulse frequency (15 Hz), average power (0.60 W), pulse duration (25 µs), and peak power (800 W).

Activated irrigation was conducted in three cycles of 20 s according to the clinical final irrigation protocol: the first cycle included 5 mL of 3% NaOCl, followed by 5 mL of ethylenediaminotetraacetic acid (EDTA) in the second cycle, and 5 mL NaOCl in the last cycle. After each subsequent irrigant, the remains from the canal were aspirated.

Group 2. Diode laser (*n* = 10)

Root canals were irradiated in three cycles of 20 s with intervals of 10 s using a 975 nm diode laser with a 200 μm fiber tip (Laser HF, HAGER&WERKEN, Duisburg, Germany): peak power = 2 W, time on = 5 ms, time off = 25 ms. The fiber tip was placed 2 mm from the apical foramen and then moved circularly along the dentinal walls towards the coronal part of the root canal.

Group 3. CI (*n* = 10)

A 31 G side-vented needle (Steri Tips, DiaDent, Burnbay, BC, Canada) was placed in each root canal 2 mm from the WL. The root canals were irrigated according to the clinical final irrigation protocol: 30 s of NaOCl (5 mL) followed by 60 s of EDTA (5 mL), then 30 s of NaOCl (5 mL). After each subsequent irrigant, the remains from the canal were aspirated.

Group 4. Control Group, C (*n* = 10)

No final disinfection protocol was conducted.

After the final disinfection protocol had been completed, the residual irrigant was aspirated, and the canals were dried with the corresponding paper points (R50 Reciproc Blue, VDW GmbH, Munich, Germany). To ensure sufficient moisture for the premixed sealer to set, the canal was dried with the paper points until the tip remained slightly moist. Then the premixed CSBS (BioRoot Flow, Septodont, Saint Maur Des Fosses, France) was introduced into the coronal part of the canal with a suitable cannula. The corresponding gutta-percha point was slowly introduced in the canal up to the WL, allowing the sealer to gently distribute along the dentinal walls. The coronal excess of the gutta-percha point in the access cavity was cut off with a heated plugger, and then the filling was vertically condensed with a cold plugger size #2 (Buchanan Hand Pluggers, Kerr, Germany).

### 2.3. Micro-CT Scanning and Analysis

Tooth specimens were scanned after instrumentation and two weeks after root canal obturation using an industrial micro-computed tomography system (Nikon XT H 225, Tokyo, Japan). To ensure adequate X-ray penetration and high-quality volumetric reconstruction, scanning was performed at 120 kV and 100 µA, with an exposure time of 500 ms. For each scan, 2880 projections were acquired during specimen rotation, resulting in reconstructed datasets with an isotropic voxel size of 14 µm. This represents the geometric resolution, resulting from the applied geometrical magnification of ~9 and detector pixel size of 127 µm. Similar X-ray parameters and specimen positioning were maintained for scans of each specimen, in order to maintain comparable X-ray penetration for scans before and after root canal obturation.

Micro-CT image analysis was performed using Volume Graphics VG Studio Max software (version 2.4). The same surface determination algorithm—locally adaptive threshold—was applied uniformly to all specimens to maintain measurement consistency between the two scanning stages. For each tooth, the corresponding before and after datasets were aligned using a best-fit (least squares method) registration algorithm, enabling accurate superposition of the same specimen for subsequent volumetric assessment.

Following registration, the initial root canal volume and the post-obturation filling material volume were quantified. The percentage of void volume within the canal was calculated as 100 − [(filling material volume × 100)/pre-filling canal volume]. The results were expressed as the percentage of canal volume that remained unfilled after obturation. The operator who calculated porosity was blinded during the study.

### 2.4. Push-Out Test

Transversal dentinal discs (1 mm thick, 5 × 5 mm) were cut from the middle third of the root using a diamond disk (Isomet 1000, Buehler, IL, USA) and prepared for the push-out test. The operator who performed push-out testing (IB) was blinded during the study. CSBS dislodgement was achieved by applying force through a stainless steel plunger one size smaller than the canal until the sealer was dislodged from the root canal space. The machine (Instron Corp., Norwood, MA, USA, movement speed 0.5 mm/min) measured the dislodgement force in Newtons (N) while the BS was measured in megapascals (MPa) and calculated using the following formula:

σ (MPa) = F/2r π h,(1)

where F/N is the maximum load measured at fracture, r is the cavity radius and h is the specimen height (1 mm).

### 2.5. Scanning Electron Microscopy Analysis

Dentinal discs for scanning electron microscopy (SEM) analysis were prepared as previously described. Samples were prepared by polishing the dentinal discs with abrasive paper of decreasing granulation until a grit size of 1200. Raw samples were recorded. The analysis was performed on a field emission SEM (FE-SEM) model JSM-7000F (Jeol Ltd., Tokyo, Japan) under 200×, 500×, and 1000× magnification and 1 kV potential. Two samples per group were recorded. Four randomly selected surfaces from each sample were captured and examined, resulting in a total of eight analyzed surfaces per group. The operator was blinded during the study. Scores for each surface were assigned as follows, modified from Escobar et al. [28]: 0 (no gaps between sealer and dentin/gutta-percha), 1 (minor flaws, <1 μm between sealer and dentin/gutta-percha), 2 (many gaps, from 1 μm to 10 μm, between sealer and dentin/gutta-percha), and 3 (no adaptation between sealer and dentin/gutta-percha, with gaps >10 μm).

### 2.6. Statistical Analyses

The results from push-out testing and micro-CT analysis were analyzed using the Kruskal–Wallis test and post hoc Dunn test, with the level of significance set at 5%.

## 3. Results

### 3.1. Results from Micro-CT Analysis

Micro-CT analysis showed void formation in all samples, with no significant differences in total porosity between the groups (*p* > 0.05). The median values of voids expressed as a percentage in each test group were as follows: SWEEPS 0.3485%, DL 2.721%, CI 2.691%, C 3.877%.

**Figure 1:**
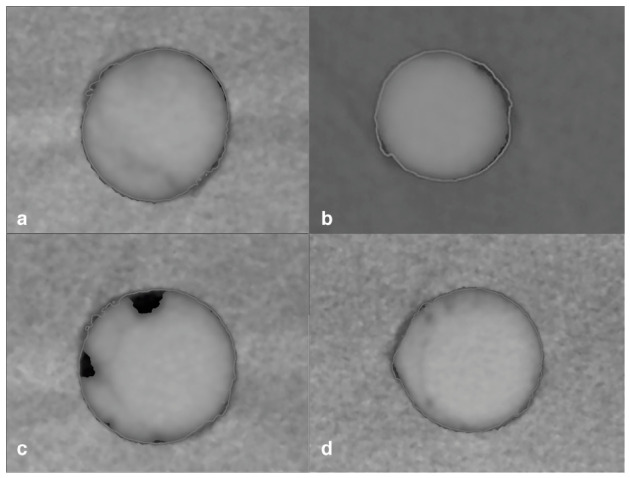
Micro-CT scans of cross-sections from the middle third of the root canal: (**a**). SWEEPS (shock wave-enhanced emission of photoacoustic streaming)group; (**b**). diode laser group; (**c**). CI (conventional irrigation) group; (**d**). control group.

Figure 1 shows micro-CT scans of cross-sections from the middle third of the root canal in each test group. Gaps between sealer and dentin were observed in all examples regardless of test group. Nevertheless, in the SWEEPS group the best adaptation between sealer and dentin can be seen, while in other figures representing DL, CI and C, a greater number and volume of voids are visible.

### 3.2. Push-Out Test Results

**Figure 2:**
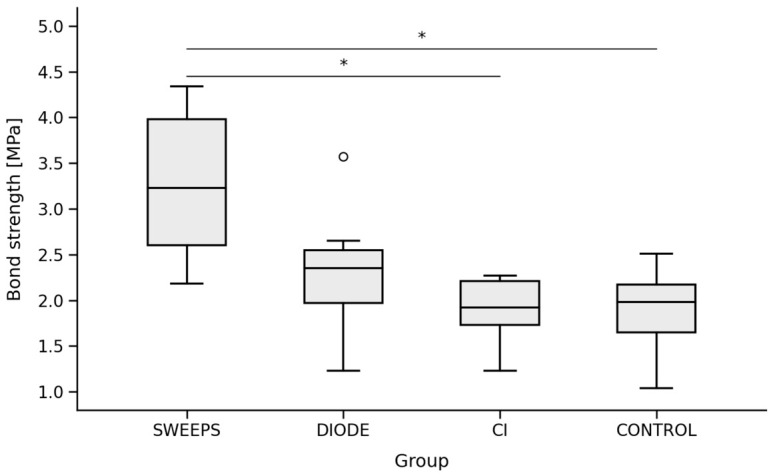
Graphical presentation of BS values of BioRoot Flow CSBS in MPa after tested disinfection protocols: SWEEPS (shock wave-enhanced emission of photoacoustic streaming), DIODE (diode laser), CI (conventional irrigation), control group. * stands for statistically significant difference between SWEEPS and diode as well as SWEEPS and control group (*p* > 0.05).

The SWEEPS group presented with the highest BS values (median 3.233 MPa), with no significant difference compared with the diode laser group (*p* > 0.05). The samples in the SWEEPS group had statistically greater bond strength compared to the samples in the CI group and C (*p* = 0.000415). There were no statistically significant differences between the diode laser group and the CI and C groups (*p* > 0.05). Table 1 shows descriptive statistics on the bond strength of CSBS after the tested disinfection protocols, expressed in MPa. Figure 2 shows a graphical presentation of the above-mentioned BS descriptive results.

### 3.3. Scanning Electron Microscopy Analysis Results

**Figure 3:**
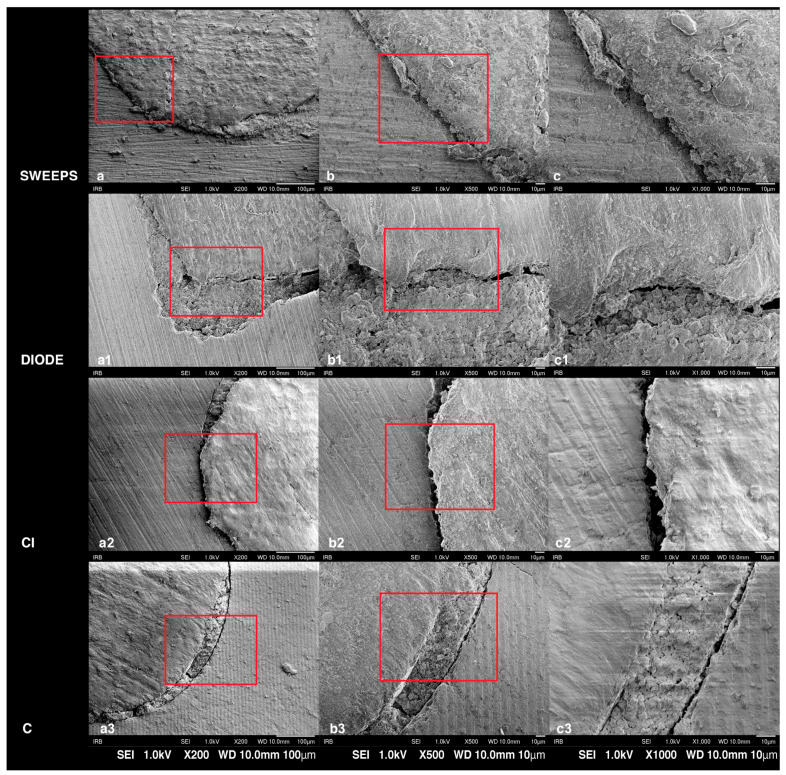
SEM analysis; an example of one examined surface in each test group, SWEEPS, diode laser, conventional irrigation (CI) and control (C), under three different magnifications: (**a**–**a3**) 200×, (**b**–**b3**) 500×, and (**c**–**c3**) 1000×. The parts of the images outlined in red within the lower-magnification images (200×, 500×) are shown in the higher-magnification SEM images (500×, 1000×).

Scanning electron microscopy was used for qualitative and descriptive analysis and visualization. The SWEEPS group scored 0 on most inspected surfaces, with 6/8 surfaces scored as 0 (no gaps between sealer and dentin/gutta-percha), while the diode laser and CI groups scored 1, with 7/8 and 6/8 surfaces, respectively, scored as 1 (minor flaws between sealer and dentin/gutta-percha). The control group showed many gaps and scored 2, with 5/8 surfaces scored as 2. Figure 3 shows an example of one randomly selected examined surface in each group under three different magnifications (200×, 500×, 1000×). Gaps are visible in each surface regardless of the test group, especially between the sealer and dentin. The SWEEPS group shows the best adaptation between the sealer and dentin, although in the example shown in Figure 3, a minor gap between the sealer and gutta-percha is visible under the highest magnification (1000×).

## 4. Discussion

The quality of root canal filling is most often estimated according to material homogeneity and the BS between the sealer and root canal dentin. Our study evaluated the porosity of a new CSBS, BioRoot Flow, after different laser-assisted protocols using a single-cone obturation technique in round canals. Regarding choosing the filling technique to ensure a tight junction between the dentinal walls and sealing material, there are no differences between the single-cone technique and the thermomechanical compaction technique in terms of the marginal gap when the root canal shape is rounded, but favorable results of thermomechanical compaction are noticed when the canal shape is oval, as stated by Zanza et al. [29]. Regarding material homogeneity in our study, porosity was observed in all tested samples, and micro-CT showed no statistical differences (*p* > 0.05) in the total void formation of BioRoot Flow CSBS after different disinfection protocols, namely two laser-assisted protocols (SWEEPS and diode laser), as well as a CI protocol; thus, the null hypothesis was accepted. In all groups, the total porosity was under 4%, which is consistent with the results of other studies [30,31,32]. Inada et al. [32] compared the porosity of CSBS to that of AH plus and found that void formation in all groups was under 5%. A study by Nguyen et al. [30] observed less than 3% total voids when comparing CSBS with silicone bioactive glass-based and epoxy resin-based root canal sealers. The porosity was calculated after 30 and 60 days, which indicates the time dependence of CSBSs. In our study, the tests were done after 2 weeks, which possibly explains why the porosity showed slightly higher values. The porosity in all described studies was assessed by micro-CT. However, in our study, we compared different disinfection protocols in combination with the same CSBS (BioRoot Flow), and only in the SWEEPS group was the median percentage of voids less than 1% (0.3485%). Furthermore, the findings of Joshi et al. [33] showed that irradiation with an Er:YAG laser lowers the contact angle between CSBS and dentin, which could possibly have an effect on the BS and may also support further testing. Although laser-assisted protocols have been tested in various fields in recent years, to our knowledge there are no papers on the influence of laser on CSBS void formation. The benefits of further studies could be clinically important, especially for the treatment of root canals with morphological anomalies.

Unlike void formation and porosity, the push-out BS has been analyzed more often in combination with different activated irrigation protocols. The results of our study showed that the SWEEPS laser-assisted protocol was beneficial with regard to the BS of the premixed CSBS (BioRoot Flow), which rejects the null hypothesis. The SWEEPS group outperformed CI (*p* < 0.05), but the diode laser group did not statistically differ from CI (*p* > 0.05). Although there was no statistical difference between the SWEEPS and diode groups, SWEEPS showed higher median BS values (3.23 MPa) than the diode group (2.34 MPa), which aligns with the results of micro-CT analysis. Peker et al. [25] showed that sonic activation methods improve the BS of CSBS. Regarding laser-activated methods and BS, Bogari et al. [26] reported enhanced BS of CSBS after Nd:YAG laser treatment, and Bago et al. [34] showed that diode laser irradiation improves the dislocation resistance of a CSBS (BioRoot RCS). All results align with the findings of our study and support activation methods for enhancing the BS of bioceramic sealer. Despite the above-mentioned results, the beneficial results of existing studies should be further examined due to the lack of information regarding the BS of CSBS after activation methods, especially laser-assisted ones.

Although our study used SEM scanning for visualization and descriptive analysis, the results also correspond with other scientific data. Mahdi & Talabani [35] confirmed the beneficial effect of activated irrigation methods, including diode laser irradiation, on bioceramic sealer penetration using SEM and confocal laser scanning microscopy (CLSM) analysis.

One of the limitations of this study relates to the setting procedure of CSBS, which is specific due to its humidity requirements, and clinical conditions that cannot be recreated ex vivo, which may affect the results and, thus, their clinical applicability. Furthermore, considering the trend toward better results in the SWEEPS group and the findings of other studies, future examination should be conducted after a longer setting period [30]. For more than a decade, methodological problems of the push-out test have been pointed out. De-Deus [36] questions the use of 1 mm thin sliced specimens for testing root filling materials with two interfaces (core material and sealer), especially when ranking different materials, but nevertheless makes the point that push-out can contribute to understanding the properties of specific filling materials and their relationship to root dentin, which is exactly what this study aimed for. In addition, research showed that despite these limitations, the push-out test is still a suitable testing tool [37]. Moreover, the aim of this study was to evaluate the effect of different laser-assisted protocols rather than the material itself, and the protocols in all test groups were conducted under the same conditions.

The clinical significance of these results lies in proposing a protocol that can enhance the bonding ability of the premixed CSBSs and decrease their porosity, allowing the full potential of biomimetic root canal sealers to be reached.

## 5. Conclusions

The BS of BioRoot Flow was higher after SWEEPS and diode laser disinfection protocols compared to CI. Voids in the CSBS single-cone filling were recorded after all disinfection treatments.

## Figures and Tables

**Table 1:** Descriptive statistics on bond strength of BioRoot Flow in MPa after tested disinfection protocols: SWEEPS, DIODE (diode laser), CI (conventional irrigation), C (control group).

Groups	*n*	Minimum	25th Percentile	Median	75th Percentile	Maximum
SWEEPS	10	2.1890	2.598	3.233	3.974	4.339
DIODE	10	1.2340	1.978	2.349	2.554	3.567
CI	10	1.2340	1.735	1.923	2.211	2.269
C	10	1.0330	1.659	1.989	2.169	2.508

## Data Availability

The original contributions presented in this study are included in the article.

## Abbreviations

The following abbreviations are used in this manuscript:

BS	Bond strength
CSBS	Calcium silicate-based sealer
C	Control group
CBCT	Cone-beam computed tomography
CI	Conventional irrigation
DL	Diode laser
EDTA	Ethylenediaminetetraacetic acid
Er:YAG	Erbium:yttrium–aluminum–garnet
FE-SEM	Field emission scanning electron microscopy
MPa	MegaPascals
NaOCl	Sodium hypochlorite
PIPS	Photon-Induced Photoacoustic Streaming
SEM	Scanning electron microscopy
SWEEPS	Shock wave-enhanced emission photoacoustic streaming
WL	Working length
